# Promoter hypermethylation of SFRP1 is an allele fraction-dependent prognostic biomarker in metastatic pancreatic ductal adenocarcinoma

**DOI:** 10.3389/fonc.2025.1568386

**Published:** 2025-05-26

**Authors:** Benjamin Stubbe, Malene P. Stoico, Simone K. Terp, Poul H. Madsen, Søren Lundbye-Christensen, Carsten P. Hansen, Laurids Ø. Poulsen, Louise S. Rasmussen, Mette N. Yilmaz, Lars H. Jensen, Torben F. Hansen, Per Pfeiffer, Anders C. Larsen, Henrik B. Krarup, Inge S. Pedersen, Jane P. Hasselby, Astrid Z. Johansen, Inna M. Chen, Julia S. Johansen, Ole Thorlacius-Ussing, Stine D. Henriksen

**Affiliations:** ^1^ Department of Gastrointestinal Surgery, Aalborg University Hospital, Aalborg, Denmark; ^2^ Department of Clinical Medicine, Aalborg University, Aalborg, Denmark; ^3^ Clinical Cancer Research Center, Aalborg University Hospital, Aalborg, Denmark; ^4^ Department of Molecular Diagnostics, Aalborg University Hospital, Aalborg, Denmark; ^5^ Unit of Research Data and Biostatistics, Aalborg University Hospital, Aalborg, Denmark; ^6^ Department of Surgery, Copenhagen University Hospital - Rigshospitalet, Copenhagen, Denmark; ^7^ Department of Oncology and Clinical Cancer Research Center, Aalborg University Hospital, Aalborg, Denmark; ^8^ Department of Oncology, University Hospital of Southern Denmark, Vejle, Denmark; ^9^ Department of Medical Oncology, Odense University Medical Center, University of Odense, Odense, Denmark; ^10^ Department of Pathology, Copenhagen University Hospital – Rigshospitalet, Copenhagen, Denmark; ^11^ Department of Oncology, Copenhagen University Hospital – Herlev and Gentofte, Herlev, Denmark; ^12^ Department of Medicine, Copenhagen University Hospital-Herlev and Gentofte Hospital, Herlev, Denmark; ^13^ Department of Clinical Medicine, Faculty of Health and Medical Sciences, University of Copenhagen, Copenhagen, Denmark

**Keywords:** epigenetics, pancreatic ductal adenocarcinoma, Sfrp1, methylation, biomarker, liquid biopsy

## Abstract

**Introduction:**

Metastatic pancreatic ductal adenocarcinoma (PDAC) is highly lethal. Promoter hypermethylation of SFRP1 (phSFRP1) in cell-free DNA is an established prognostic biomarker in PDAC. We used digital droplet PCR (ddPCR) to examine whether the prognostic impact of phSFRP1 was allele fraction (AF) dependent.

**Methods:**

Prospectively collected plasma samples were analyzed blinded. Dual-strand methylation ddPCR assays were designed for SFRP1, with single-strand assay for the reference gene EPHA3. Patients were stratified into unmethylated SFRP1 (umSFRP1), low phSFRP1 AF (phSFRP1^low^), and high phSFRP1 AF (phSFRP1^high^). Survival was assessed with Kaplan–Meier curves. The 3-, 6-, and 12-month absolute risk difference (ARD) was calculated, and performance assessed with ROC analyses.

**Results:**

Overall, 354 patients were included. Patients with umSFRP1 (*n*=137) had a mOS of 9.1 months compared to 7.2 months in phSFRP1^low^ (*n*=78) and 3.4 months in phSFRP1^high^ (*n*=143, *P*<0.01). phSFRP1^high^ was associated with increased mortality at 3 (ARD 26%, 95%CI: 15, 37), 6 (ARD 37%, 95%CI: 26, 48), and 12 months (ARD 23%, 95%CI: 14, 33). phSFRP1^low^ was associated with increased mortality at 12 months (ARD 13%, 95%CI: 2, 25) but not at 3 (ARD -3%, 95%CI: -13, 8) or 6 months (ARD 3%, 95%CI: -10, 17). phSFRP1 significantly improved performance in predicting mortality compared to only clinical variables (AUC: 0.70-0.71 *vs*. 0.54-0.57).

**Discussion:**

Patients with phSFRP1^high^ had significantly shorter survival than phSFRP1^low^ or umSFRP1, indicating AF-dependent prognostic effects. phSFRP1^low^ had a worse prognosis than umSFRP1 at only 12 months, indicating dynamic changes. This could help personalize the treatment of PDAC.

## 1 Introduction

With a 5-year survival of only 12% across all stages and 3% in patients with metastatic disease, pancreatic ductal adenocarcinoma (PDAC) continues to be one of the most lethal malignancies worldwide (1). PDAC has been estimated to become the second leading cause of cancer-related death by 2026 (2). For patients with metastatic disease, the only treatment option is palliative chemotherapy (3). In real-world data from Denmark (2011-2016), the median overall survival (mOS) of metastatic PDAC ranged from 8.2 months in patients treated with FOLFIRINOX (folinic acid, 5-FU, irinotecan, oxaliplatin) to 4.1 months in patients treated with gemcitabine monotherapy (4). More accurate prognostic tools are needed to help guide personalized treatment choices and improve patient outcomes (5, 6). The only routinely used biomarker in patients with PDAC is plasma levels of sialy Lewis carbohydrate antigen 19-9 (CA19-9). However, its use is limited by low sensitivity, being expressed in other diseases, and lack of production in approximately 10% of the Caucasian population (7, 8).

Liquid biopsies could offer a minimally invasive, real-time snapshot of tumor alterations, allowing for dynamic monitoring of therapy response and disease progression (9).

Aberrant DNA methylation of gene promoters is a key molecular event driving tumorigenesis in various cancers, including PDAC (10). These epigenetic alterations are detectable in cell-free DNA (cfDNA) and can promote cancer growth and progression by silencing tumor suppressor genes (9, 11, 12).

A pathway of particular relevance is the Wnt/β-catenin signaling pathway, a key regulator of numerous processes, such as proliferation, apoptosis, differentiation, and cancer cell stemness (13–15). Pancreatic carcinogenesis relies heavily on Wnt signaling (16). The Wnt/β-catenin pathway regulates nuclear β-catenin concentration through the destruction complex built from adenomatosis polyposis coli (APC), glycogen synthase 3 β, and axin proteins (17). In the absence of negative modulation, Wnt binds to the cysteine-rich domain of the Frizzled receptor, which leads to the activation of disheveled, inactivation of the destruction complex, and finally, accumulation of β-catenin (17).

An essential negative modulator of this pathway is secreted frizzled-related protein 1 (SFRP1), which inhibits Wnt either by directly binding to the Wnt ligand, by binding to the Frizzled receptor and thus preventing the binding of the Wnt ligand, or by directly binding cytoplasmic β-catenin (17, 18). Reduced expression of SFRP1 in tumor tissue has been proposed as a potential prognostic biomarker in several cancers, including PDAC (18, 19). SFRP1 is primarily downregulated through promoter hypermethylation (20, 21).

While most studies have examined SFRP1 in tumor tissue or cell lines, recent research has demonstrated the utility of a cfDNA-based liquid biopsy approach for examining promoter hypermethylation of *SFRP1* (phSFRP1) and assessing prognosis in patients with PDAC (22–24).

However, these studies were limited by using only semiquantitative methylation analysis. This study aimed to address this limitation by employing a fully quantitative droplet digital PCR (ddPCR) methodology to identify whether a high phSFRP1 allele fraction (AF) in patients with stage IV PDAC impacts prognosis more than low or no phSFRP1 AF.

## 2 Materials and methods

### 2.1 Patients

This study was a retrospective, blinded analysis of patients with histologically verified stage IV PDAC. Patients were treated with best supportive care (BSC) or 1. line palliative treatment with FOLFIRINOX (5-FU, irinotecan, oxaliplatin), gemcitabine, gemcitabine and capecitabine, gemcitabine and nab-paclitaxel, or gemcitabine, nab-paclitaxel and tocilizumab (an antibody against the IL-6 receptor). Patient data and pretreatment plasma samples were obtained from the BIOPAC study (“BIOmarkers in patients with PAncreatic Cancer (BIOPAC) - can they provide new information of the disease and improve diagnosis and prognosis of the patients”; ClinicalTrials.gov ID NCT03311776). The BIOPAC study is a prospective national Danish multicenter open cohort study enrolling patients presenting with pancreatic cancer before surgical or chemotherapeutic treatment. Patients were consecutively included at seven Danish hospitals between July 2008 and October 2020. Clinical data was not received until methylation analysis was completed. Some patients received gemcitabine, nab-paclitaxel, and tocilizumab as part of the PACTO trial (ClinicalTrials.gov ID NCT02767557).

The original BIOPAC study protocol was approved by the Danish Ethics Committee (VEK, j.nr. KA-20060113) and the Danish Data Protection Agency (j.nr. 2012-58-0004; HGH-2015-027; I-Suite j.nr. 03960; and PACTICUS P-2020-834). The current study was approved by the Regional Research Ethics Committee of Northern Denmark (approval number: N-20130037). The study was carried out following the Reporting Recommendations for Tumor Marker Prognostic Studies (REMARK) guidelines, and the study was conducted by the Danish Law of Research Ethics, based on the Declaration of Helsinki.

### 2.2 Methylation analysis

#### 2.2.1 Design and optimization of methylation-specific droplet digital PCR assays

Methylation-specific ddPCR primers and probes were designed to target the *SFRP1* promoter previously investigated (22, 25, 26). To increase the sensitivity, dual-strand droplet digital PCR assays were designed using Beacon Designer 8.21. An assay against a non-CpG-containing part of *EPHA3* was used as an internal control for estimating the total cfDNA concentration (27).

Primers and probes were manufactured by TAG Copenhagen. Primer and probe sequences are displayed in **Supplementary Table 1**.

#### 2.2.2 DNA isolation and bisulfite treatment

EDTA plasma was centrifuged at 2300 g for 10 min at 4°C and stored at -80°C. Upon thawing, the plasma was centrifuged at 12,000 × g for 10 minutes at 4°C. cfDNA was extracted from 0.5–2 mL plasma on the QIAsymphony (Qiagen) using the DSP Circulating DNA Kit (Qiagen). The isolated cfDNA was eluted in 60 μL elution buffer and stored in DNA Lobind tubes (Eppendorf) at −20°C (<2 weeks). DNA was evaporated to 20 µL using low-temperature vacuum centrifugation (SAVANT DNA120 SpeedVac Concentrator) and sodium bisulfite-converted using an EZ-96 DNA Methylation-Direct™ MagPrep kit (Zymo Research) according to the manufacturer’s instructions. When conducting the bisulfite conversion, one methylation-positive control (commercially available *in vitro* methylated DNA; Zymo Research), two different unmethylated controls (commercially available *in vitro* nonmethylated DNA; Zymo Research; Qiagen), and a no template control (nuclease-free water) were included. The bisulfite-converted cfDNA samples were analyzed using ddPCR immediately after bisulfite conversion or stored at −20°C until use (< 2 weeks).

#### 2.2.3 Droplet digital PCR

The methylation status of the *SFRP1* promoter region was analyzed using the QX200™ Droplet Digital™ PCR System (ddPCR, Bio-Rad). The ddPCR mixture consisted of 1x ddPCR Supermix for Probes (Bio-Rad), 1.36 μM of each SFRP1 primer, 227 nM of each SFRP1 probe, 909 nM of each EPHA3 primer, 284 nM EPHA3 probe, and bisulfite-converted DNA, in a final volume of 22 μl. Droplets were generated using the Automated Droplet Generator (Bio-Rad). The PCR was performed in a C1000 Touch Thermal Cycler (Bio-Rad) at 95°C for 10 min, 50 cycles of 94°C for 1 min and 55°C for 2 min, followed by 98°C for 10 min, with a ramp rate of 1°C/s. Following PCR, samples were incubated at either 4°C for at least 30 min or at 12°C for a minimum of 4 h (<24 h). Every ddPCR plate included a methylation-positive control (commercially available *in vitro* methylated DNA; Zymo Research), two different unmethylated controls (commercially available *in vitro* nonmethylated DNA; Zymo Research; Qiagen), and a no template control (nuclease-free water).

#### 2.2.4 Data analysis

The fluorescence data for all individual droplets were analyzed platewise using QX Manager Software version 1.2 (Bio-Rad). A minimum of 10,000 accepted droplets was required for further analysis. Thresholds were manually set using positive and negative controls with gating based on fluorescence amplitude in 1D and 2D plots. Based on the fraction of positive droplets in each well, concentrations (copies per well) of methylated DNA were calculated by the software. The target gene concentrations included the signal from both sense and antisense strands, compared to a single strand in the control concentration. Thus, a normalized AF was calculated by dividing the target gene’s concentration by twice the control’s concentration.

### 2.3 Statistical methods

An optimal AF cutoff for patients with detectable phSFRP1 was determined using maximally selected rank statistics implemented in the “maxstat” R package. Patients were classified into three groups based on phSFRP1 AF: high AF of phSFRP1 (phSFRP1^high^), low AF of phSFRP1 (phSFRP1^low^), and unmethylated SFRP1 (umSFRP1). Fisher’s exact test was used to compare categorical variables. As most of the continuous variables were highly non-normal, the Kruskal–Wallis test was used to compare continuous variables, and the Spearman correlation coefficient was used to evaluate correlation between variables.

Survival was calculated from the time of the pretreatment blood sample until either death from any cause or the end of follow-up on January 8, 2023. Data were treated as time-to-event data and analyzed with established survival analysis methods. As the proportional hazard assumption was violated, comparisons were quantified using absolute risk differences (ARD). These risks were determined using pseudo-observations in combination with generalized linear regression analysis (28). To reflect the different survival risks, time points 3, 6, and 12 months were selected preemptively. The standard error, *P* values, and confidence intervals were calculated using robust variance estimation.

Univariable models were fitted for SFRP1 methylation status and the covariates age > 65, sex (male or female), Eastern Cooperative Oncology Group (ECOG) performance status (PS) > 1, treatment (best supportive care, gemcitabine, gemcitabine + capecitabine, gemcitabine + nab-paclitaxel, gemcitabine + nab-paclitaxel + tocilizumab, or FOLFIRINOX) and CA19-9 (below or above 860). The univariable models were supplemented with a multivariable model including all variables. As there is no consensus on the cutoff value for CA19–9 to optimize the prognostic value, the value of 860 was chosen based on a previous study by our group (23). Supplemental analyses were also performed using various other cutoffs proposed in the literature (23).

Survival was illustrated using Kaplan–Meier survival curves supplemented with log-rank tests. ROC analyses were performed with tenfold cross-validation to evaluate if the addition of AF-dependentSFRP1 methylation improved performance in predicting mortality.

A *P* value < 0.05 was considered statistically significant, and 95% confidence intervals were used where applicable. Statistical calculations were performed in Stata v. 17 (StataCorp) or R version 4.2.2 (R Foundation for Statistical Computing).

## 3 Results

Plasma samples were obtained from 369 patients presenting with metastatic PDAC. Four patients were excluded due to an insufficient number of droplets. Additionally, 11 patients were excluded upon receival of clinical data, as they had started palliative chemotherapy before sampling. Thus, 354 patients were included in the study.

### 3.1 Sample and patient characteristics

In total, phSFRP1 was detectable in 217 patients and undetectable in 137 patients. The median number of accepted droplets was 20.386 (interquartile range (IQR): 19.712, 20.908). The median concentration of phSFRP1 in samples with detectable levels was 124 copies/ml (IQR: 19, 875). The reference gene *EPHA3* was detected in all samples, with a median concentration of 2.900 (IQR: 1.554, 7.151) copies/ml. The median AF of phSFRP1 among samples with detectable phSFRP1 was 1.58% (IQR: 0.32%, 5.54%). Maximally selected rank statistics were employed to determine an optimal phSFRP1 AF cutoff of 0.53%.

According to this cutoff, patients were stratified into three groups: no detectable phSFRP1 (umSFRP1), detectable phSFRP1 with a phSFRP1 AF below 0.53% (phSFRP1^low^), and detectable phSFRP1 with a phSFRP1 AF above 0.53% (phSFRP1^high^). Of the 217 patients with detectable phSFRP1, 143 were stratified into the phSFRP1^high^ group and 74 into the phSFRP1^low^ group (**Supplementary Figure 1**).

Patients with phSFRP1^high^ were younger (median 66 years) than those with umSFRP1 or phSFRP1^low^ (71 years, *P =* 0.01). Plasma CA19–9 and ECOG PS were significantly higher in patients with either phSFRP1^high^ or phSFRP1^low^ compared to those in patients with umSFRP1 (*P <* 0.01 and *P =* 0.01). A moderate positive correlation was observed between phSFRP1 AF and CA 19–9 levels (Rho = 0.32, *P <* 0.01), and a weaker but statistically significant correlation was observed between phSFRP1 AF and ECOG PS (Rho = 0.12, *P* < 0.03). Conversely, phSFRP1 AF was negatively correlated with patients age (Rho = -0.15, *P* < 0.01). Patients with phSFRP1^high^ and phSFRP1^low^ were more likely to have a tumor located in the cauda and metastases in the liver than umSFRP1 patients (*P <* 0.01 and *P <* 0.01, respectively). There were no differences in BMI, sex, or treatment among the three groups (**Table 1**). The cohort comprised 285 patients of Caucasian ethnicity, 1 of African descent and 4 of other ethnic backgrounds. Ethnicity data were unavailable for 64 patients due to the absence of ethnicity registration at one of the seven inclusion sites.

**Table 1:** Baseline characteristics of stage IV patients with PDAC according to SFRP1 methylation status.

Characteristics	All	umSFRP1	phSFRP1^low^	phSFRP1^high^	*P* value
	(*n* = 354)	(*n* = 137)	(*n* = 74)	(*n* = 143)	
**Age, years (median, IQR)**	69 (62-74)	71 (64-74)	71 (65-76)	66 (60-72)	0.01^a^
**CA19-9, kU/L (median, IQR)**	2590 (218-17564)	695 (74-4821)	2700 (281-7136)	8170 (574-67900)	*P* < 0.01^a^
**BMI (median, IQR)**	24 (21.3-26.7)	24 (21.1-26.7)	24 (21.6-27.1)	23 (21.5-26.6)	0.81^a^
**Sex**					0.34^b^
Male	208 (59%)	74 (54%)	47 (64%)	87 (61%)	
Female	146 (41%)	63 (46%)	27 (36%)	56 (39%)	
ECOG Performance Status					*P* < 0.01^b^
0	108 (31%)	45 (33%)	19 (26%)	44 (31%)	
1	186 (53%)	75 (55%)	46 (62%)	65 (45%)	
2	45 (13%)	10 (7%)	7 (9%)	28 (20%)	
3	3 (1%)	1 (1%)	2 (3%)	0 (0%)	
Unknown	12 (3%)	6 (4%)	0 (0%)	6 (4%)	
Treatment					0.43^b^
Best supportive care	17 (5%)	4 (3%)	1 (1%)	12 (8%)	
Gemcitabine	149 (42%)	55 (40%)	33 (45%)	61 (43%)	
Gem + cap	22 (6%)	8 (6%)	6 (8%)	8 (6%)	
Gem + nab	102 (29%)	39 (28%)	24 (32%)	39 (27%)	
Gem + nab + tocilizumab	21 (6%)	10 (7%)	4 (5%)	7 (5%)	
FOLFIRINOX	43 (12%)	21 (15%)	6 (8%)	16 (11%)	
Location of the primary tumor					*P* < 0.01^b^
Caput	166 (47%)	64 (47%)	46 (62%)	56 (39%)	
Corpus	93 (26%)	46 (34%)	12 (16%)	35 (24%)	
Cauda	64 (18%)	15 (11%)	15 (20%)	34 (24%)	
Diffuse	19 (5%)	9 (7%)	1 (1%)	9 (6%)	
Papil	2 (1%)	0 (0%)	0 (0%)	2 (1%)	
Unknown	10 (3%)	3 (2%)	0 (0%)	7 (5%)	
Location of metastasis					*P* < 0.01^b^
Liver	193 (55%)	58 (42%)	39 (53%)	96 (67%)	
Lung	20 (6%)	11 (8%)	6 (8%)	3 (2%)	
Carcinosis	30 (8%)	25 (18%)	1 (1%)	4 (3%)	
Other	16 (5%)	7 (5%)	5 (7%)	4 (3%)	
Multiple	95 (27%)	36 (26%)	23 (31%)	36 (25%)	

Data are n (%) unless otherwise specified. umSFRP1, unmethylated SFRP1; phSFRP1, promoter hypermethylation of SFRP1; phSFRP1^low^, phSFRP1 AF < 0.53%; phSFRP1^high,^ phSFRP1 AF > 0.53%. Gem + cap, gemcitabine and capecitabine; Gem + nab, gemcitabine and nab-paclitaxel; Gem + nab + tocilizumab, Gemcitabine, nab-paclitaxel and tocilizumab; FOLFIRINOX, 5-FU, irinotecan and oxaliplatin.

a Kruskal–Wallis one-way analysis of variance.

b Fisher’s Exact Test. CA19–9 was missing in 3 patients.

### 3.2 phSFRP1 as an allele fraction-dependent prognostic marker

**Figure 1:**
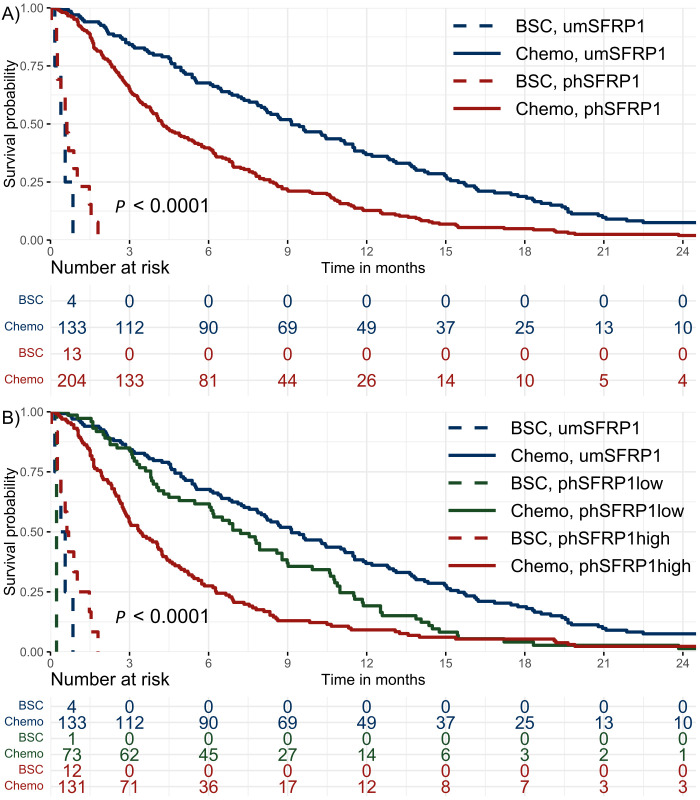
Kaplan–Meier survival distributions of patients with stage IV PDAC. Patients were stratified according to SFRP1 promoter methylation status and initial treatment with either best supportive care or chemotherapy. The risk table shows the number of patients at risk in 3-month intervals. **(A)** Dichotomized analysis of methylation status. umSFRP1, no detectable phSFRP1; phSFRP1, any detectable phSFRP1 AF. **(B)** SFRP1 methylation status grouped by phSFRP1 AF. umSFRP1, no detectable phSFRP1; phSFRP1^low^, phSFRP1 AF < 0.53%; phSFRP1^high^, phSFRP1 AF > 0.53%.

The 6- and 12-month survival rates were 48% and 21%, respectively. Fourteen patients were alive at the end of follow-up, with a minimum follow-up of 24.6 months. The mOS of chemotherapy-treated patients with any detectable phSFRP1 AF was 4.2 months compared to 9.1 months in umSFRP1 (**Figure 1A**). The mOS of chemotherapy-treated patients with phSFRP1^high^ was 3.4 months compared to 7.2 months in patients with phSFRP1^low^ (**Figure 1B**). The 3-month mortality among chemotherapy-treated patients with umSFRP1 and phSFRP1^low^ was approximately equal at 16% and 15%, respectively.

**Figure 2:**
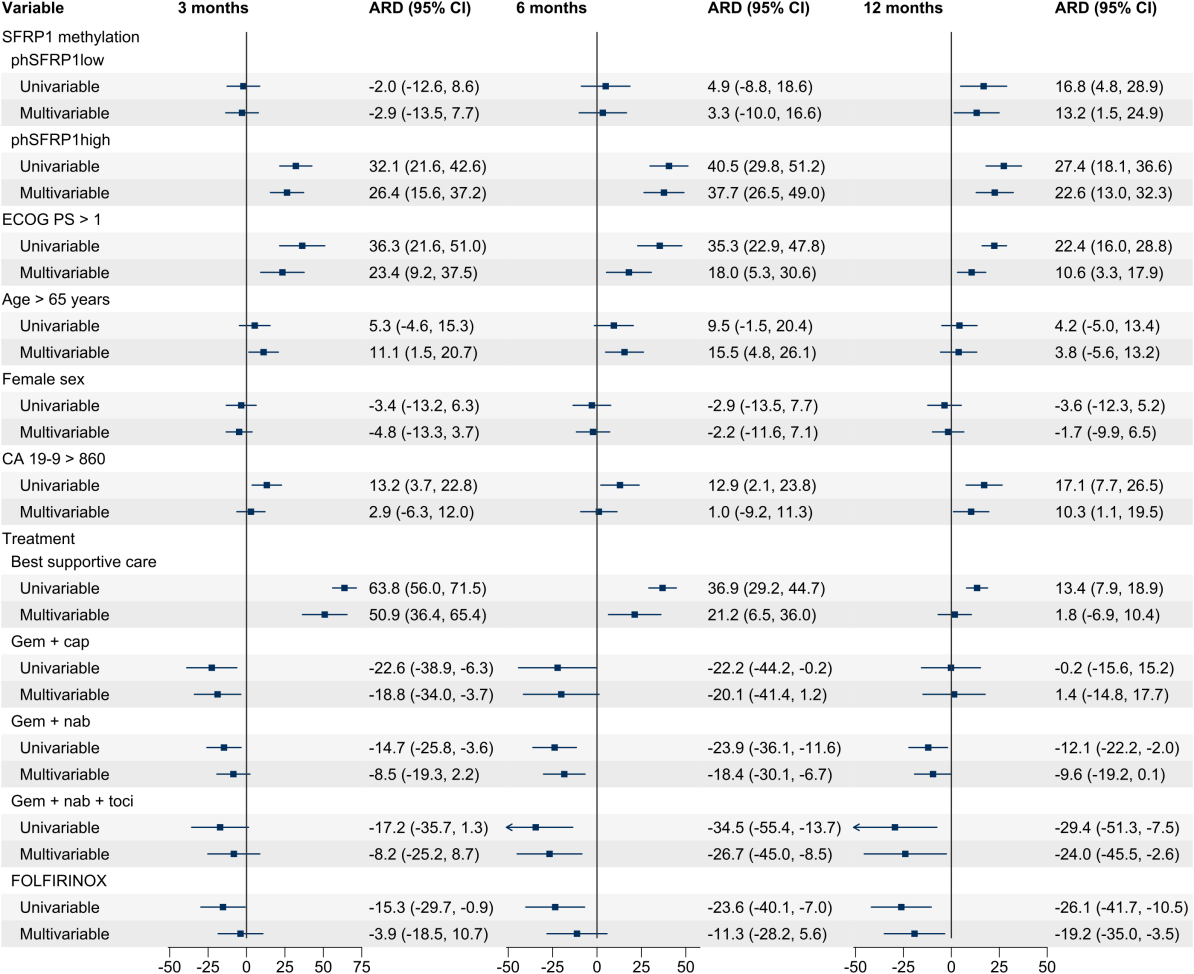
Univariable and multivariable ARD for patients with stage IV PDAC. A positive number indicates an increased risk of death, and a negative number indicates a lower risk of death. Analyses were performed for 3, 6, and 12 months according to SFRP1 promoter methylation status and covariates. Cases with missing data were excluded from the analysis. phSFRP1^low^, phSFRP1 AF below 0.53%; phSFRP1^high^, phSFRP1 AF above 0.53%; Gem + cap, gemcitabine and capecitabine; Gem + nab, gemcitabine and nab-paclitaxel; Gem + nab + toci, gemcitabine, nab-paclitaxel, and tocilizumab. The references were umSFRP1, ECOG PS ≤ 1, age ≤ 65, male sex, CA19-9 ≤ 860, and treatment with gemcitabine monotherapy.

Univariable regression models revealed phSFRP1^high^ to be significantly associated with a higher mortality risk at 3 months (absolute risk difference (ARD) 32.1%, 95% CI: 21.6, 42.6), 6 months (ARD 40.5%, 95% CI: 29.8, 51.2), and 12 months (ARD 27.4%, 95% CI: 18.1, 36.6) when compared to that of umSFRP1 (**Figure 2**). The significant association was confirmed for all time points when adjusting for ECOG PS, age, sex, CA19-9, and type of chemotherapy (3-month ARD 26.4%, 95% CI: 15.6, 37.2; 6-month ARD 37.7%, 95% CI: 26.5, 49.0; and 12-month ARD 22.6%, 95% CI: 13.0, 32.3). The increased risk associated with phSFRP1^high^ was either comparable to or higher than that of the known prognostic factors ECOG PS > 1, CA19–9 levels, and type of chemotherapy.

In chemotherapy-treated patients, those with phSFRP1^high^ had substantially shorter survival compared to that of patients with phSFRP1^low^ or umSFRP1, regardless of the type of chemotherapy (**Supplementary Figure 3**). In contrast, among BSC-treated patients with phSFRP1^high^ had an mOS of 0.6 months, compared to 0.4 months in those with umSFRP1.

Among gemcitabine-treated patients, there was no difference in survival between those with umSFRP1 and phSFRP1^low^.

In univariable regression models, phSFRP1^low^ was significantly associated with a higher risk of death at 12 months (ARD 16.8%, 95% CI: 4.8, 28.9) but not at 3 months (ARD -2.0%, 95% CI: -12.6, 8.6) or 6 months (ARD 4.9%, 95% CI: -8.8, 18.6). This was confirmed for all time points when adjusting for ECOG PS, age, sex, CA19-9, and type of chemotherapy (3-month ARD -2.9%, 95% CI: -13.5, 7.7; 6-month ARD 3.3%, 95% CI: -10.0, 16.6; 12-month ARD 13.2%, 95% CI: 1.5, 24.9).

The multivariable model did not initially include tumor location or location of metastasis. However, given the significant difference in distribution according to SFRP1 methylation, their impact was subsequently assessed (**Table 1**). Adding these variables to the multivariable model did not affect the association between phSFRP1 and ARD (**Supplementary Figure 2**).

**Figure 3:**
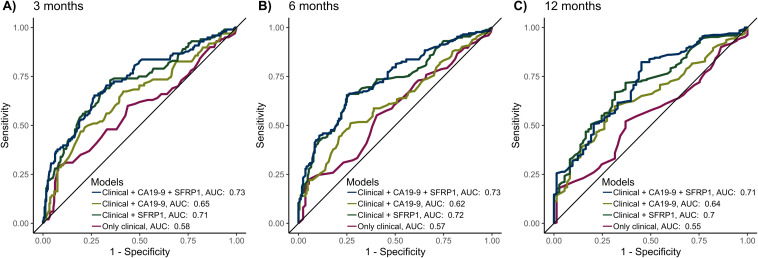
The predictive performance of the models for 3-, 6-, and 12-month mortality, validated by 10-fold cross-validation. The 4 models included the following variables: 1) Only the clinical variables age > 65, sex, and ECOG PS > 1. 2) Clinical variables as well as CA19-9 > 860. 3) Clinical variables as well as AF-dependent SFRP1 methylation status (umSFRP1, phSFRP1^low^ or phSFRP1^high^). 4) Clinical variables as well as CA19-9 > 860 and AF-dependent SFRP1 methylation status. Cases with missing data were excluded from the analysis. **(A)** Performance of models at 3 months. **(B)** Performance of models at 6 months. **(C)** Performance of models at 12 months.

ROC curves were computed to assess whether adding SFRP1 methylation status improved the performance of regression models in predicting 3-, 6-, and 12-month mortality (**Figure 3**).

Generally, the models including only clinical variables (age > 65, sex, and ECOG PS > 1) had the least predictive power, with an area under the curve (AUC) of predicting mortality between 0.55-0.58. The addition of AF-dependent SFRP1 methylation status (umSFRP1, phSFRP1^low^, or phSFPR1^high^) led to significantly better predictive power (AUC 0.7-0.72, *P <* 0.01 at all time points). Adding CA19–9 to clinical variables also improved the predictive power, with an AUC between 0.62-0.65 (3 months, *P <* 0.01; 6 months, *P =* 0.07; and 12 months, *P <* 0.01). Models containing phSFRP1, CA19-9, and clinical variables had the highest power (AUC 0.71-0.73) and were significantly better than models with CA19–9 and clinical variables (3 months, *P <* 0.01; 6 months, *P <* 0.01; and 12 months, *P =* 0.02). Analyses were also carried out using several other cutoffs for CA19–9 previously described in literature (23). The addition of AF-dependent SFRP1 methylation status to a model of clinical variables and CA19–9 significantly increased performance across all time points and all CA19–9 cut-offs (**Supplementary Figure 4**).

**Figure 4:**
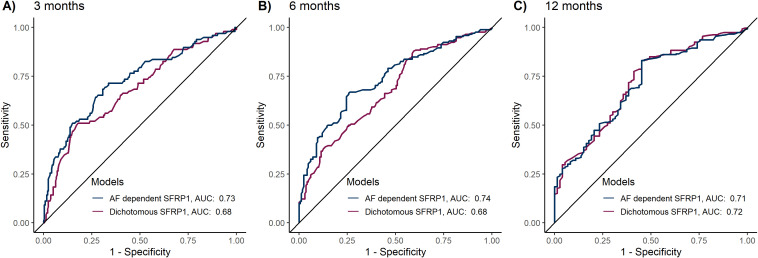
Comparative predictive performance of dichotomous versus allele fraction-dependent SFRP1 methylation analysis. The predictive models included the variables age > 65, sex, ECOG PS > 1, CA19-9 > 860 and either dichotomous SFRP1 methylation status (umSFRP1 or phSFRP1) or allele fraction-dependent SFRP1 methylation analysis (umSFRP1, phSFRP1^low^, or phSFRP1^high^). Performance was assessed at 3-, 6-, and 12 months, and models were computed using 10-fold cross-validation. **(A)** Performance of models at 3 months. **(B)** Performance of models at 6 months. **(C)** Performance of models at 12 months.

Lastly, we examined if the AF-dependent SFRP1 methylation analysis improved predictive power compared to the previous dichotomous approach. The AF-dependent approach demonstrated significantly superior predictive performance at 3 months (AUC 0.73 *vs*. 0.68, *P* = 0.03) and 6 months (AUC 0.74 *vs*. 0.68, *P* < 0.01), **Figure 4**. No significant difference was observed at 12 months (AUC 0.72 *vs* 0.71, *P =* 0.48).

## 4 Discussion

This study demonstrated that phSFRP1^high^, measured in plasma cfDNA, was associated with a worse prognosis than phSFRP1^low^ or umSFRP1. To our knowledge, this is the first study to demonstrate an AF-dependent prognostic impact of phSFRP1 in metastatic PDAC.

The patient cohort was divided into three groups according to the level of SFRP1 methylation. Subsequently, the association of SFRP1 methylation with mortality risk was evaluated using regression models for 3-, 6-, and 12-month mortality. phSFRP1^high^ was consistently associated with an increased mortality risk at all time points compared to that of umSFRP1 and phSFRP1^low^. This increased risk was either greater than or similar to the mortality risk of the known prognostic factors ECOG PS, CA19-9, and type of chemotherapy. Notably, there were no considerable changes in the effect sizes of phSFRP1^high^ in the multivariable model.

In contrast, phSFRP1^low^ was only significantly associated with increased mortality risk at 12 months compared to that of umSFRP1, but not at 3 or 6 months. The effect sizes were considerably smaller than those of phSFRP1^high^. These results demonstrate the clinical utility of phSFRP1 measured by plasma cfDNA as a prognostic biomarker for patients with stage IV PDAC and indicate a clinically meaningful difference in prognostic impact depending on phSFRP1 AF.

During the initial 3 months, the survival of patients with phSFRP1^low^ mirrored that of patients with umSFRP1, after which their mortality rates gradually increased, being comparable to phSFRP1^high^ by 12 months. This finding could suggest a time-dependent influence of phSFRP1 on PDAC progression and highlights the potentially dynamic nature of this epigenetic alteration throughout the course of the disease. This finding could indicate the utility of this biomarker as a dynamic tool for disease progression or response to therapy. However, further investigation is needed, as a recent small cohort found no differences in SFRP1 methylation frequency after treatment (29).

Chemotherapy-treated patients with phSFRP1^high^ had shorter survival than patients with phSFRP1^low^ or umSFRP1 irrespective of chemotherapy type. This was not observed among BSC-treated patients, although the small sample size limits interpretability. Previous studies indicate that the effects of phSFRP1 differ among BSC-treated patients (23, 24). This difference in effect according to treatment could indicate that phSFRP1 is a predictive biomarker of chemotherapy efficacy. Reduced SFRP1 expression and high β-catenin expression have previously been linked to chemotherapy resistance (30–32). Furthermore, a recent study on metastatic breast cancer found that patients with SFRP1 alterations in ctDNA responded poorly to endocrine therapy plus placebo but had increased benefit of endocrine therapy plus the CDK4/6 inhibitor ribociclib (33). Further research is needed to ascertain the predictive value of phSFRP1 in PDAC.

Achieving improvements (or stability) in patient quality of life with chemotherapy is closely correlated with survival improvements (34). Given their extremely poor prognosis, it is possible that some proportion of patients with phSFRP1^high^ did not benefit from treatment with chemotherapy. In contrast, the better prognosis among patients with umSFRP1 could suggest additional benefit from treatment, but further research is needed to confirm this hypothesis. A cfDNA-based phSFRP1 analysis could be an additional tool to aid clinicians and patients in finding the optimal balance between quantity and quality of life.

In previous studies, a dichotomous methylation analysis was employed due to limitations of the quantitative PCR methodology, necessitating a preamplification and limiting the quantitative aspects of the technique (22–24, 26). Comparatively, the ddPCR methodology allows for complete quantification of SFRP1 methylation status without requiring preamplification. Furthermore, it allows for the simultaneous quantification of a reference gene to estimate phSFRP1 AF. Normalization of the target material is important for preventing either the under- or overestimation of methylation levels, as DNA quality and integrity can vary when using clinical samples (27, 35). The gene EPHA3 was chosen as a reference, as it has previously been demonstrated to be a robust control (27).

Despite forgoing preamplification, the ddPCR methodology appears to be more sensitive than the previous methodology. In this cohort, phSFRP1 was detected in approximately 61% of patients, compared to the 45-50% of comparable patients with detected phSFRP1 demonstrated in previous studies (22, 23). The rate of methylation in this cohort more closely matches rates previously demonstrated in PDAC tissue (64-70%) (19, 20, 36). Additionally, the AF-dependent SFRP1 methylation analysis yielded significantly better predictive performance compared to the dichotomous approach. This finding indicates that the absolutely quantitative ddPCR approach is both more reliable and confers clinically meaningful information not captured by the dichotomous approach. However, the dichotomous qPCR approach could remain a valid option in resource-poor settings where equipment to perform ddPCR may not be available.

phSFRP1 is potentially a target for several methods of targeted treatments. First, demethylating treatments could reverse the promoter hypermethylation of SFRP1. Both activation of the Wnt/β-catenin pathway and loss of SFRP1 expression have been linked to chemotherapy resistance (30–32, 37, 38). Cell studies suggest higher sensitivity to chemotherapy and less aggressive characteristics following demethylating treatment of cell lines with phSFRP1 (30, 38–40). However, the risk of activating latent oncogenes with globally demethylating treatments may limit the use of this approach (41). Second, it may be possible to mimic the effects of the SFRP1 protein. A recent study identified a mimetic compound inhibiting cell growth in phSFRP1 cells by downregulating the phosphorylated LRP6 receptor (42). However, this therapeutic approach is at a very early stage. Third, an oral drug inhibiting traf2 and Nck-interacting kinase (TNIK) has been shown to suppress growth and increase apoptosis in colon cancer cells through Wnt signaling inhibition (43). TNIK is essential for colon cancer growth and tumor initiation (44). While TNIK has been associated with poor prognosis in PDAC, evidence suggests that oncogenic mechanisms may differ according to cancer type (45). The potential role of Wnt inhibition through TNIK inhibition requires further investigation in PDAC.

Patients with phSFRP1^high^ had significantly higher levels of CA19–9 and worse PS than patients with umSFRP1 or phSFRP1^low^. Additionally, patients with phSFRP1^high^ were significantly younger than patients with umSFRP1 or phSFRP1^low^. Younger patients may be more likely to have a more aggressive tumor subtype, leading to worse PS by time of diagnosis and higher release of CA19-9. Unfortunately, additional tissue for analysis was not available for further investigation. There was a weak but significant correlation between CA19–9 and phSFRP1 AF. As the inclusion of addition of phSFRP1 significantly improved model performance irrespective of CA19–9 cutoff, the effects of phSFRP1 are unlikely to represent a surrogate for CA19-9.

Notably, including these factors in multivariable regression models did not impact the effects of phSFRP1. Furthermore, in ROC analysis, performance in predicting mortality was significantly improved by adding SFRP1 methylation status, indicating that phSFRP1 contributes prognostic information distinct from known prognostic factors. Interestingly, the performance of models including only clinical factors was moderate at all time points. These results further support the potential clinical utility of phSFRP1 as a prognostic biomarker.

Retrospective studies are potentially at risk of selection bias, and poor registration of outcomes and variables could bias statistical analyses. However, these risks are partially alleviated by the prospective collection and registration of data in the BIOPAC study, which was conducted to develop new blood-based biomarkers. Furthermore, there was no censoring in the study period, and the methylation analysis was blinded to clinical data. Liquid biopsies are inherently limited by their reliance on the tumor to release sufficient DNA into the plasma to be detectable. However, metastatic PDAC has been shown to release high amounts of circulating tumor DNA (46). Furthermore, phSFRP1 has been demonstrated to be detectable and prognostic in patients with stage I-II PDAC (24). Last, the patient population was included from all Danish centers treating patients with PDAC, ensuring a cohort likely to be representative of the Danish population of patients with metastatic PDAC eligible for palliative chemotherapy.

In conclusion, this study emphasizes the clinical potential of phSFRP1 in cfDNA as a prognostic biomarker for stage IV PDAC. Our findings indicate that the degree of phSFRP1 AF significantly correlates with patient prognosis and could indicate the effect of chemotherapy. This finding indicates a substantial benefit of the ddPCR-based methodology, offering a more nuanced approach than previous dichotomous analyses of phSFRP1. Furthermore, our results indicate a time-dependent effect of SFRP1, which warrants further investigation. These results could enhance patient stratification and personalize the treatment of patients with stage IV PDAC.

## Data Availability

The raw data supporting the conclusions of this article will be made available by the authors, without undue reservation.

## References

[1] Siegel RL Miller KD Wagle NS Jemal A. Cancer statistics, 2023. CA Cancer J Clin. (2023) 73:17–48. doi: 10.3322/caac.21763 36633525

[2] Rahib L Wehner MR Matrisian LM Nead KT. Estimated projection of US cancer incidence and death to 2040. JAMA Netw Open. (2021) 4:1–14. doi: 10.1001/jamanetworkopen.2021.4708 PMC8027914 33825840

[3] Conroy T Pfeiffer P Vilgrain V Lamarca A Seufferlein T O’Reilly EM. Pancreatic cancer: ESMO Clinical Practice Guideline for diagnosis, treatment and follow-up. Ann Oncol. (2023) 34(11):987–1002. doi: 10.1016/j.annonc.2023.08.009 37678671

[4] Rasmussen LS Fristrup CW Jensen BV Pfeiffer P Weber B Yilmaz MK. Initial treatment and survival in 4163 Danish patients with pancreatic cancer: A nationwide unselected real-world register study. Eur J Cancer. (2020) 129:50–9. doi: 10.1016/j.ejca.2020.01.015 32120275

[5] Mizrahi JD Surana R Valle JW Shroff RT. Pancreatic cancer. Lancet. (2020) 395:2008–20. doi: 10.1016/S0140-6736(20)30974-0 32593337

[6] Jiang Y Sohal DPS. Pancreatic adenocarcinoma management. JCO Oncol Pract. (2023) 19:19–32. doi: 10.1200/OP.22.00328 36137253

[7] Von Rosen A Linder S Harmenberg U Pegert S. Serum levels of CA 19–9 and CA 50 in relation to lewis blood cell status in patients with Malignant and benign pancreatic disease. Pancreas. (1993) 8:160–5. doi: 10.1097/00006676-199303000-00004 8460090

[8] Goonetilleke KS Siriwardena AK. Systematic review of carbohydrate antigen (CA 19-9) as a biochemical marker in the diagnosis of pancreatic cancer. Eur J Surg Oncol. (2007) 33:266–70. doi: 10.1016/j.ejso.2006.10.004 17097848

[9] Alix-Panabières C Pantel K. Liquid biopsy: from discovery to clinical application. Cancer Discov. (2021) 11:858–73. doi: 10.1158/2159-8290.CD-20-1311 33811121

[10] Esteller M. Cancer epigenomics: DNA methylomes and histone-modification maps. Nat Rev Genet. (2007) 8:286–98. doi: 10.1038/nrg2005 17339880

[11] Brancaccio M Natale F Falco G Angrisano T. Cell-free dna methylation: The new frontiers of pancreatic cancer biomarkers’ discovery. Genes. (2020) 11:1–16. doi: 10.3390/genes11010014 PMC7017422 31877923

[12] Baylin SB Jones PA. Epigenetic determinants of cancer. Cold Spring Harb Perspect Biol. (2016) 8:a019505. doi: 10.1101/cshperspect.a019505 27194046 PMC5008069

[13] Wen X Wu Y Awadasseid A Tanaka Y Zhang W. New advances in canonical wnt/β-catenin signaling in cancer. Cancer Manag Res. (2020) 12:6987–98. doi: 10.2147/CMAR.S258645 PMC7418153 32821165

[14] Zhan T Rindtorff N Boutros M. Wnt signaling in cancer. Oncogene. (2017) 36:1461–73. doi: 10.1038/onc.2016.304 PMC5357762 27617575

[15] MacDonald BT Tamai K He X. Wnt/β-catenin signaling: components, mechanisms, and diseases. Dev Cell. (2009) 17:9–26. doi: 10.1016/j.devcel.2009.06.016 19619488 PMC2861485

[16] Zhang Y Morris JP Yan W Schofield HK Gurney A Simeone DM. Canonical wnt signaling is required for pancreatic carcinogenesis. Cancer Res. (2013) 73:4909–22. doi: 10.1158/0008-5472.CAN-12-4384 PMC3763696 23761328

[17] MacDonald BT He X. Frizzled and LRP5/6 receptors for wnt/-catenin signaling. Cold Spring Harb Perspect Biol. (2012) 4:a007880–a007880. doi: 10.1101/cshperspect.a007880 23209147 PMC3504444

[18] Baharudin R Tieng FYF Lee LH Mutalib NSA. Epigenetics of SFRP1: The dual roles in human cancers. Cancers. (2020) 12:1–20. doi: 10.3390/cancers12020445 PMC7072595 32074995

[19] Jiang N Hu MG Tang WB Yin ZZ Wang ZZ Liu Q. Aberrant loss of SFRP1 expression is associated with poor prognosis in pancreatic cancer. Int J Clin Exp Pathol. (2017) 10:6064–70.

[20] Bu XM Zhao CH Zhang N Gao F Lin S Dai XW. Hypermethylation and aberrant expression of secreted fizzled-related protein genes in pancreatic cancer. World J Gastroenterol. (2008) 14:3421–4. doi: 10.3748/wjg.14.3421 PMC2716598 18528941

[21] Hattori N Sako M Kimura K Iida N Takeshima H Nakata Y. Novel prodrugs of decitabine with greater metabolic stability and less toxicity. Clin Epigenetics. (2019) 11:111. doi: 10.1186/s13148-019-0709-y 31370878 PMC6670186

[22] Stubbe BE Henriksen SD Madsen PH Larsen AC Krarup HB Pedersen IS. Validation of SFRP1 promoter hypermethylation in plasma as a prognostic marker for survival and gemcitabine effectiveness in patients with stage IV pancreatic adenocarcinoma. Cancers. (2021) 13:5717. doi: 10.3390/cancers13225717 34830873 PMC8616084

[23] Stubbe BE Madsen PH Larsen AC Krarup HB Pedersen IS Hansen CP. Promoter hypermethylation of SFRP1 as a prognostic and potentially predictive blood-based biomarker in patients with stage III or IV pancreatic ductal adenocarcinoma. Pancreatology. (2023) 23:512–21. doi: 10.1016/j.pan.2023.05.003 37230892

[24] Stubbe BE Larsen AC Madsen PH Krarup HB Pedersen IS Lundbye-Christensen S. Promoter hypermethylation of SFRP1 as a prognostic and potentially predictive blood-based biomarker in patients with localized pancreatic ductal adenocarcinoma. Front Oncol. (2023) 13:1211292. doi: 10.3389/fonc.2023.1211292 37333823 PMC10272559

[25] Henriksen SD Madsen PH Larsen AC Johansen MB Drewes AM Pedersen IS. Cell-free DNA promoter hypermethylation in plasma as a diagnostic marker for pancreatic adenocarcinoma. Clin Epigenetics. (2016) 8:1081–91. doi: 10.1016/j.pan.2021.05.003 PMC5112622 27891190

[26] Henriksen SD Stubbe BE Madsen PH Johansen JS Jensen BV Hansen CP. Cell-free DNA promoter hypermethylation as a diagnostic marker for pancreatic ductal adenocarcinoma – An external validation study. Pancreatology. (2021) 21:1081–91. doi: 10.1016/j.pan.2021.05.003 33994313

[27] Pharo HD Andresen K Berg KCG Lothe RA Jeanmougin M Lind GE. A robust internal control for high-precision DNA methylation analyses by droplet digital PCR. Clin Epigenetics. (2018) 10:24. doi: 10.1186/s13148-018-0456-5 29484034 PMC5822558

[28] Andersen PK Pohar Perme M. Pseudo-observations in survival analysis. Stat Methods Med Res. (2010) 19:71–99. doi: 10.1177/0962280209105020 19654170

[29] García-Ortiz MV Cano-Ramírez P Toledano-Fonseca M Cano MT Inga-Saavedra E Rodríguez-Alonso RM. Circulating NPTX2 methylation as a non-invasive biomarker for prognosis and monitoring of metastatic pancreatic cancer. Clin Epigenetics. (2023) 15:118. doi: 10.1186/s13148-023-01535-4 37481552 PMC10362605

[30] Ren J Wang R Song H Huang G Chen L. Secreted frizzled related protein 1 modulates taxane resistance of human lung adenocarcinoma. Mol Med. (2014) 20:164–78. doi: 10.2119/molmed.2013.00149 PMC4002848 24643460

[31] Bernemann C Hülsewig C Ruckert C Schäfer S Blümel L Hempel G. Influence of secreted frizzled receptor protein 1 (SFRP1) on neoadjuvant chemotherapy in triple negative breast cancer does not rely on WNT signaling. Mol Cancer. (2014) 13:1–12. doi: 10.1186/1476-4598-13-174 25033833 PMC4110378

[32] Zhang Q Meng XK Wang WX Zhang RM Zhang T Ren JJ. The Wnt/β-catenin signaling pathway mechanism for pancreatic cancer chemoresistance in a three-dimensional cancer microenvironment. Am J Transl Res. (2016) 8:4490–8. PMC5095343 27830034

[33] André F Su F Solovieff N Hortobagyi G Chia S Neven P. Pooled ctDNA analysis of MONALEESA phase III advanced breast cancer trials. Ann Oncol. (2023) 34(11):1003–14. doi: 10.1016/j.annonc.2023.08.011 37673211

[34] Kristensen A Vagnildhaug OM Grønberg BH Kaasa S Laird B Solheim TS. Does chemotherapy improve health-related quality of life in advanced pancreatic cancer? A systematic review. Crit Rev Oncol Hematol March. (2016) 99:286–98. doi: 10.1016/j.critrevonc.2016.01.006 26819138

[35] So AP Vilborg A Bouhlal Y Koehler RT Grimes SM Pouliot Y. A robust targeted sequencing approach for low input and variable quality DNA from clinical samples. NPJ Genomic Med. (2018) 3:2. doi: 10.1038/s41525-017-0041-4 PMC5768874 29354287

[36] Bu X Zhao C Dai X. Aberrant expression of Wnt antagonist SFRP1 in pancreatic cancer. Chin Med J (Engl). (2008) 121:952–5. doi: 10.1097/00029330-200805020-00016 18706212

[37] Manegold P Lai KKY Wu Y Teo JL Lenz HJ Genyk YS. Differentiation therapy targeting the β-catenin/CBP interaction in pancreatic cancer. Cancers. (2018) 10:1–19. doi: 10.3390/cancers10040095 PMC5923350 29596326

[38] Wu KM Li ZQ Yi WZ Wu MH Jiang MJ Zhang Y. Restoration of secreted frizzled-related protein 1 suppresses growth and increases cisplatin sensitivity in laryngeal carcinoma cells by downregulating NHE1. Int J Clin Exp Pathol. (2017) 10:8334–43. PMC6965448 31966684

[39] Taguchi Y Iwadate M Umeyama H. SFRP1 is a possible candidate for epigenetic therapy in non-small cell lung cancer. BMC Med Genomics. (2016) 9:28. doi: 10.1186/s12920-016-0196-3 27534621 PMC4989892

[40] Cooper SJ Von Roemeling CA Kang KH Marlow LA Grebe SK Menefee ME. Reexpression of tumor suppressor, sFRP1, leads to antitumor synergy of combined HDAC and methyltransferase inhibitors in chemoresistant cancers. Mol Cancer Ther. (2012) 11:2105–15. doi: 10.1158/1535-7163.MCT-11-0873 PMC3928542 22826467

[41] Swerev TM Wirth T Ushmorov A. Activation of oncogenic pathways in classical Hodgkin lymphoma by decitabine: A rationale for combination with small molecular weight inhibitors. Int J Oncol. (2017) 50:555–66. doi: 10.3892/ijo.2016.3827 28035374

[42] Dahl E Villwock S Habenberger P Choidas A Rose M Klebl BM. White paper: mimetics of class 2 tumor suppressor proteins as novel drug candidates for personalized cancer therapy. Cancers. (2022) 14. doi: 10.3390/cancers14184386 PMC9496810 36139547

[43] Zhou K Cheong JE Krishnaji ST Ghalali A Fu H Sui L. Inhibition of wnt signaling in colon cancer cells via an oral drug that facilitates TNIK degradation. Mol Cancer Ther. (2023) 22:25–36. doi: 10.1158/1535-7163.MCT-21-0801 36302395

[44] Mahmoudi T Li VSW Ng SS Taouatas N Vries RGJ Mohammed S. The kinase TNIK is an essential activator of Wnt target genes. EMBO J. (2009) 28:3329–40. doi: 10.1038/emboj.2009.285 PMC2776109 19816403

[45] Zhang Y Jiang H Qin M Su X Cao Z Wang J. TNIK serves as a novel biomarker associated with poor prognosis in patients with pancreatic cancer. Tumor Biol January. (2016) 37:1035–40. doi: 10.1007/s13277-015-3881-5 26269113

[46] Bettegowda C Sausen M Leary RJ Kinde I Wang Y Agrawal N. Detection of circulating tumor DNA in early- and late-stage human Malignancies. Sci Transl Med. (2014) 6. doi: 10.1126/scitranslmed.3007094 PMC4017867 24553385

