# Supplementary material for: Promoter hypermethylation of SFRP1 is an allele fraction-dependent prognostic biomarker in metastatic pancreatic ductal adenocarcinoma
Source: Front Oncol. 2025 May 26;15:1568386. doi: 10.3389/fonc.2025.1568386 (PMC12146188; doi:10.3389/fonc.2025.1568386)
Supplement: Supplementary file 1 [file DataSheet1.docx]

Supplementary Material

**Supplementary Table 1:** Primer and probe sequences used for methylation analysis of SFRP1 and the reference gene.

| **Gene** | **Chromosome location** | **Genomic position (hg38)** | **DNA strand** | **Forward Primer** | **Reverse Primer** | **Probe** | **Amplicon length (bp)** |
| --- | --- | --- | --- | --- | --- | --- | --- |
| SFRP1 | 8 | 41309435 to 41309525 | Sense | GGAGTTGATTGGTTGCGC | CGCGACACTAACTCCG | Fam-CGAAACTCCTACGACCGAACCCTCG-3'-BHQ-1 | 91 |
| SFRP1 | 8 | 41309435 to 41309529 | Antisense | CGCGGTATTGATTTCGGA | GCCGAAAACTAATTAACTACGC | Fam-ACGACTCCGAAAACTCGACCGTAAA-3'-BHQ-1 | 95 |
| EPHA3* | 3 |  |  | GGATTTATTAGGTGTGTAATGTTATGGATT | ACTCCACATAAATCTTCTAAACTAAATTCCT | Hex-TTGGTTGAGAATAAATTGGGTTT-3'-BHQ-1 | 99 |

*Published in (https://clinicalepigeneticsjournal.biomedcentral.com/articles/10.1186/s13148-018-0456-5)


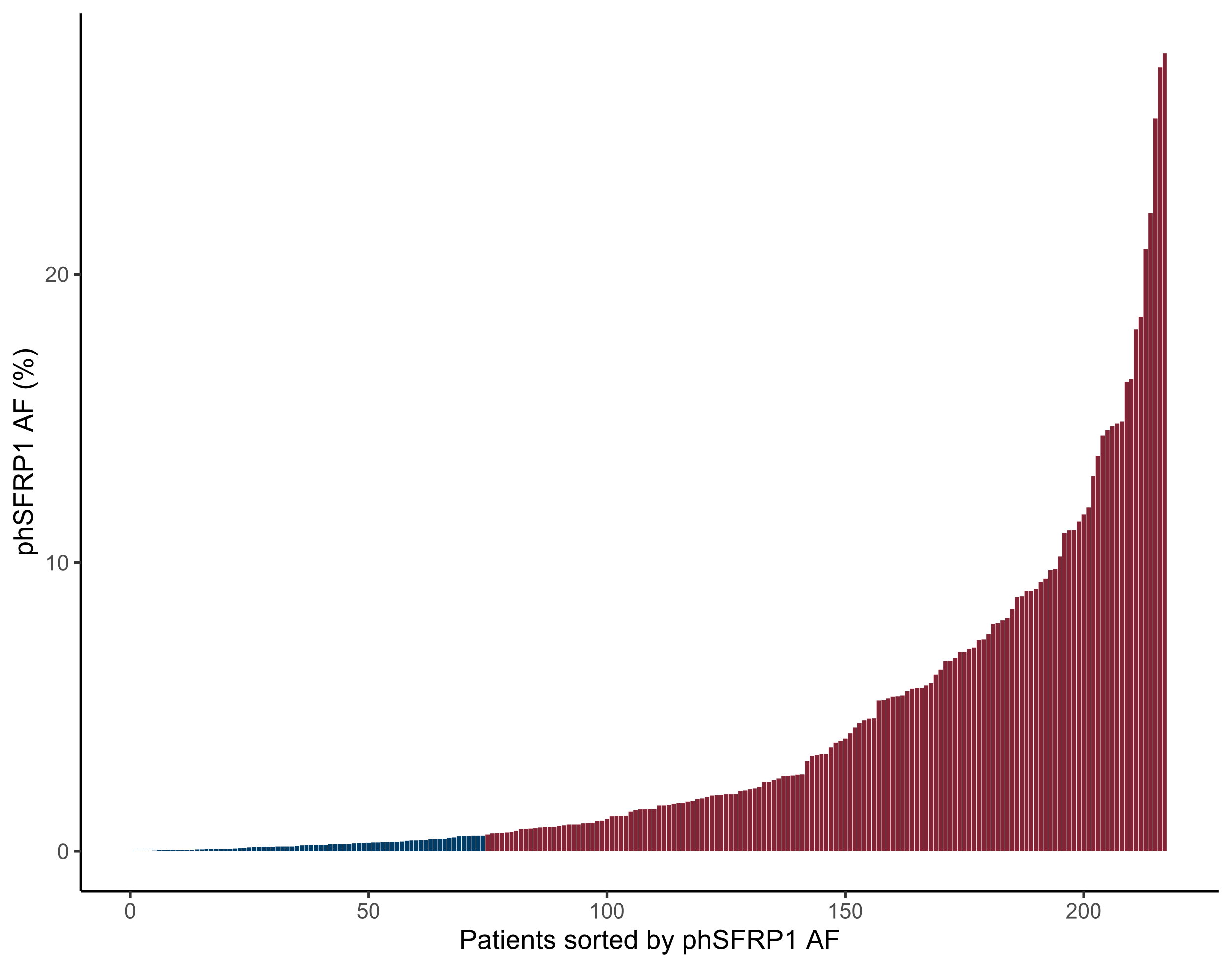


**Supplementary Figure 1.** All patients with detectable phSFRP1, sorted by phSFRP1 allele fraction. phSFRP1^low^ (blue); phSFRP1 allele fraction < 0.53%. phSFRP1^high^ (red); phSFRP1 allele fraction > 0.53%.


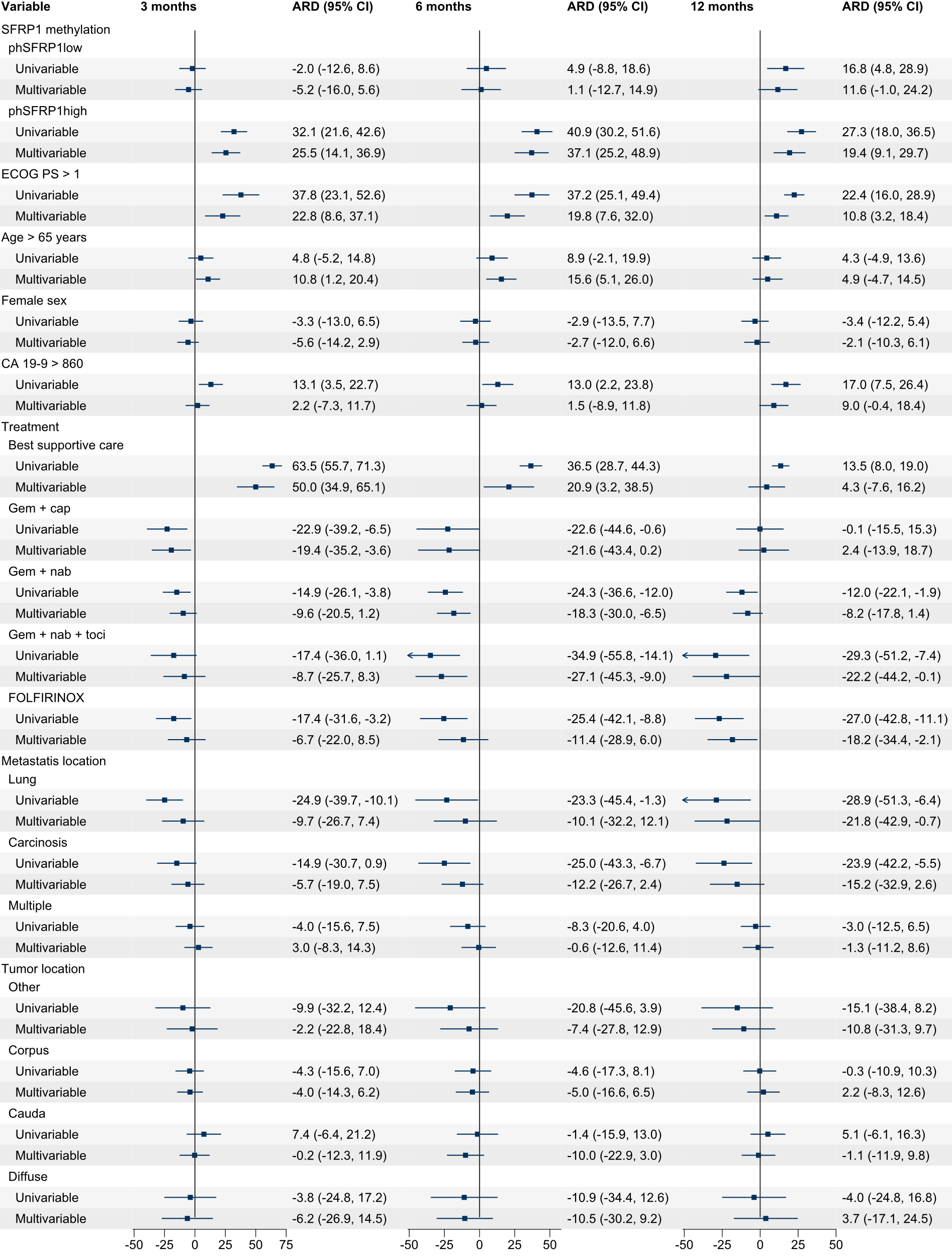


**Supplementary Figure 2.** Crude and adjusted differences in absolute risk of death for patients with stage IV PDAC. Positive number indicates increased risk of death, negative number indicates lower risk of death. Analyses performed for 3, 6 and 12 months according to SFRP1 promoter methylation status and covariates. phSFRP1^low^, phSFRP1 AF below 0.53%; phSFRP1^high^, phSFRP1 AF above 0.53%; Gem/Cap, Gemcitabine and Capecitabine; Gem/Nab, Gemcitabine and Nab-Paclitaxel; FOL, FOLFIRINOX; PACTO, Gemcitabine, Nab-Paclitaxel, and Tocilizumab. The references were the following: umSFRP1, ECOG PS ≤ 1, age ≤ 65, male sex, CA 19-9 ≤ 860, treatment with gemcitabine monotherapy, liver metastasis and primary tumor in caput.


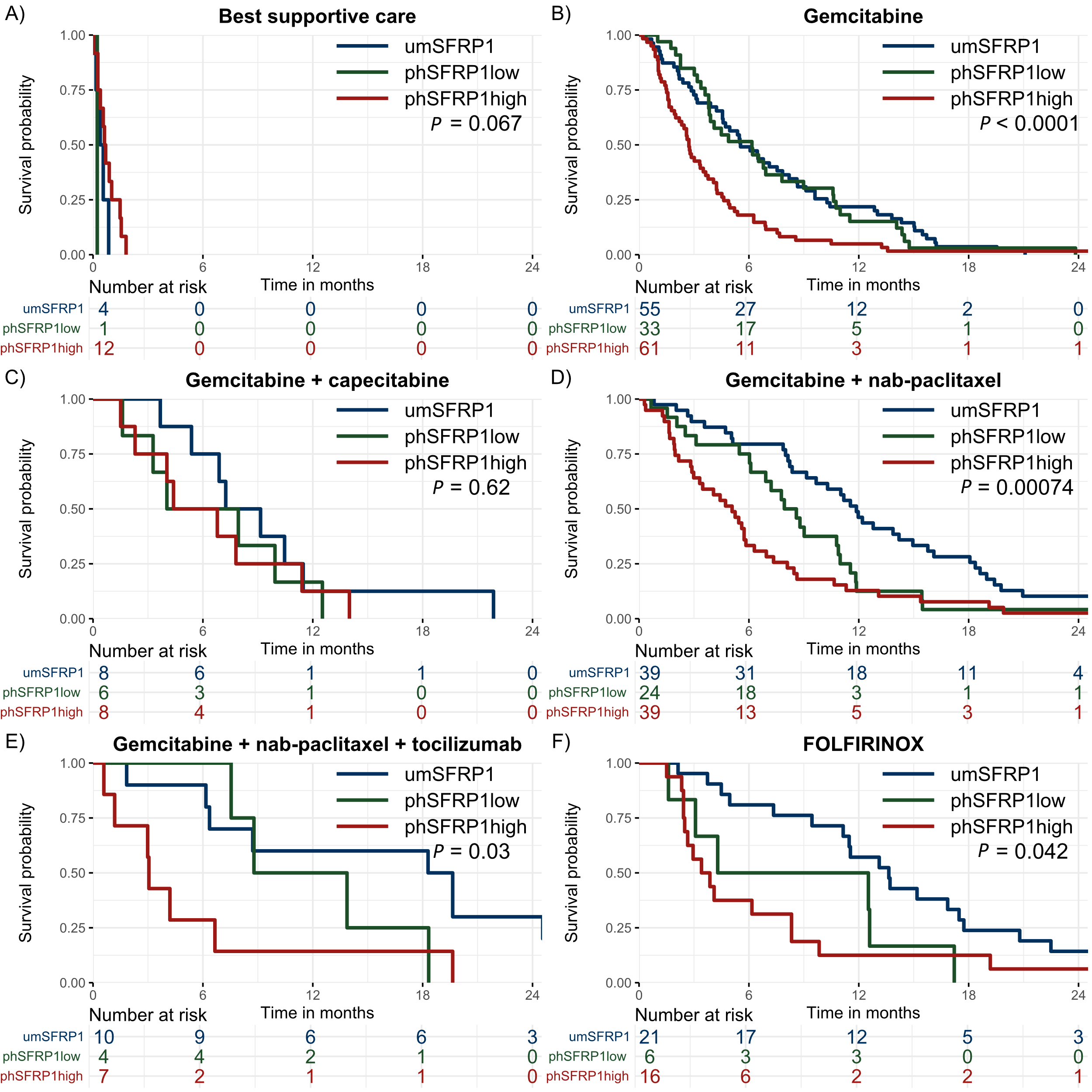


**Supplementary Figure 3.** Survival of patients with stage IV PDAC according to SFRP1 methylation status (umSFRP1, phSFRP1^low^ or phSFRP1^high^) and treatment. A) Best supportive care. B) Gemcitabine. C) Gemcitabine + capecitabine. D) Gemcitabine + nab-paclitaxel. E) Gemcitabine + nab-paclitaxel + tocilizumab. F) FOLFIRINOX.


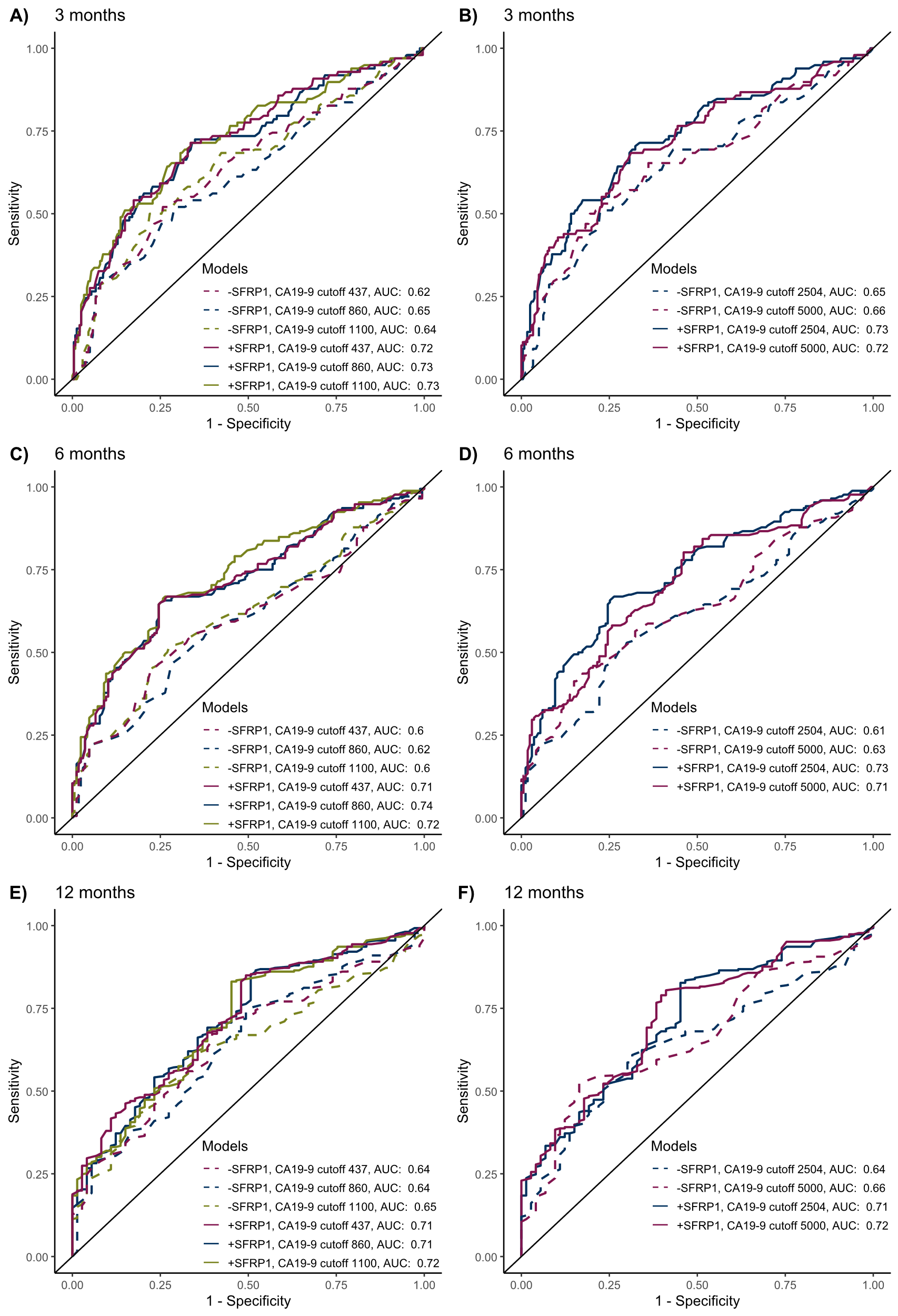


**Supplementary Figure 4.** Predictive performance of models with 10-fold validation according to SFRP1 methylation and CA19-9 cutoffs. All models include the clinical variables age > 65, sex, PS > 1, and CA19-9 at different cutoffs. +SFRP1, Addition of AF-dependent SFRP1 methylation (umSFRP1, phSFRP1^low^, phSFRP1^high^). -SFRP1, Without AF-dependent SFRP1 methylation. A) Mortality prediction at 3 months. B) Mortality prediction at 3 months. C) Mortality prediction at 12 months. D) Mortality prediction at 12 months. E) Mortality prediction at 12 months. F) Mortality prediction at 12 months.
